# Influence of Subjective Postural Vertical with Closed and Open Eyes in Patients with Hemiplegic and Pusher Behavior with Unilateral Spatial Neglect After Stroke: A Cross-Sectional Study

**DOI:** 10.3390/brainsci14111108

**Published:** 2024-10-31

**Authors:** Kota Sawa, Kazu Amimoto, Takuya Miyamoto, Miko Tamura

**Affiliations:** 1 Department of Physical Therapy, Faculty of Health Sciences, SBC Tokyo Medical University, Urayasu 279-0014, Chiba, Japan; 2 Department of Physiotherapy, Rehabilitation, Sendai Seiyo Gakuin University, Sendai 984-0022, Miyagi, Japan; k_amimoto@seiyogakuin.ac.jp; 3 Department of Rehabilitation, Takenotsuka Noshinkei Rehabilitation Hospital, Adachi 121-0064, Tokyo, Japan; taa9yaa.1116@gmail.com; 4 Department of Rehabilitation, Tums Sakura Hospital Edogawa, Edogawa 133-0063, Tokyo, Japan; miko.35miko.tam@gmail.com

**Keywords:** cognitive perception, integration, verticality, pusher behavior, unilateral spatial neglect

## Abstract

Background: When integrating visual and somatosensory processing into the subjective postural vertical using the Romberg test, patients with hemiplegic can be sorted into either post-stroke or pushers with unilateral spatial neglect (USN). This study aimed to clarify the characteristics of the integrated processing of the integrated subjective postural vertical (ISPV) with open or closed eyes in patients with hemiplegic and/or pusher with USN. Methods: This cross-sectional study included 91 patients with hemiplegic and 45 with pusher and USN. The outcomes included the ratio and sum of SPV with the eyes closed and open. Statistical analyses were performed using the parametric and/or non-parametric Wilcoxon rank-sum test, Mann–Whitney *U* test, or chi-square test after the Shapiro–Wilk test. Results: The outcomes in the 91 patients with hemiplegic were as follows: moderate-to-severe ISPV with ratio, 1.64°; ISPV sum (ISPVS), 9.41°. The outcomes in the 45 patients with pusher and USN were as follows: moderate-to-severe, ISPV: 1.35°, and ISPVS: 13.96°. No significant differences were observed between the two groups in terms of demographic data or ISPV. However, the number of patients with pusher syndrome was significantly higher in the ISPVS group than in stroke patients with hemiplegic. Conclusions: Adaptation occurs by integrating sensory modalities, and the pusher behavior in patients with USN is characterized by the specific pathophysiology of a two-modality disorder with visual and somatosensory deficits. This study provides key insights into the pathophysiological characteristics of patients with pusher syndrome and USN.

## 1. Introduction

First-time stroke affects approximately two million people in Japan per year, posing significant challenges in the fields of medicine, health, nursing, and welfare [1,2]. During rehabilitation, a comprehensive assessment, screening for cognitive evaluation, and a detailed individualized assessment are used to predict prognosis, plan subsequent treatments, and understand and interpret the pathology from the acute phase onward [3,4,5].

Human postural control is based on the relationships between visual, vestibular, and somatosensory information projected from neural network systems [6,7,8,9]. Sensory information is integrated into the parietal lobe and purposefully transmitted to the supplementary motor and prefrontal cortices for optimal motor commands [10,11,12]. Sensory integration plays an essential role in responding to and providing feedback on motor tasks and living environments, as well as in inputting and integrating various types of sensory information into the visual, somatosensory, and vestibular senses [13,14].

The Romberg test evaluates differences in body sensory information between closed and open eyes to assess the weighting and dependence of visual-somatosensory information [15,16,17]. The Romberg test is positive in patients with stroke and cerebellar disease. Visual dependence is known to be higher in patients with pusher syndrome and unilateral spatial neglect (USN) than in those with hemiplegic [18]. Patients with USN are more likely to fall because of the Romberg test and exhibit impaired predictive postural control. Increased difficulty in postural control may have a negative impact on the degree of independence in activities of daily living (ADLs). The Romberg ratio in healthy participants was 2.5, and increased Romberg ratios have been reported in patients with stroke hemiplegic and cerebellar patients [18,19]. This metric is important in evaluating the Romberg ratio, serving as an indicator of an individual’s ability to process multisensory information. However, factors affecting the ability to control posture remain unclear. To address this, we propose a new evaluation index for postural control combining SPV and SPV with eyes opened (SPV-EO) for use as a test index. It is both useful for evaluating sensory integration and important for performing an integration evaluation, similar to the use of an integrated visuomotor system in vertical cognition.

Vertical cognition refers to the ability to subjectively evaluate the body as being vertically positioned. This metric was evaluated as being integrated by the analysis of somatosensory input with the eyes either closed or open. A characteristic of postural control in patients with hemiplegic and pusher syndrome is that the postural vertical deviates when the eyes are closed. Previous studies on the recovery process of stroke patients have shown that postural verticality negatively influences the recovery of ADLs [20,21]. SPV measures coordinate to determine postural verticality in the eyes-closed posture; however, SPV with eyes open (SPV-EO) evaluates visual-somatosensory perception. It is currently unclear to what extent visual influence is present, as in the integrated evaluation. It has been suggested that this measure can clarify the extent to which somatosensory and visual factors influence each other and may be a valid mixed measure for pusher cases with USN. This is a multisensory test of integrated vertical cognition that can be defined as an evaluation of the visual-somatosensory influence of sensory information in vertical cognition, which is an evaluation of the integrated processing of sensory information of the SPV with eyes closed or opened. According to Romberg, assuming a theory that considers the influence of vision on somatosensory perception in a mathematical equation, integrated vertical cognition is processed by (EC − EO/EC + EO × 100) [15,16] and can capture biases from sensory information in patients with hemiplegic and pusher behaviors with USN. However, as the “ratio” cannot detect errors that combine visual and somatosensory information, in the present study, we evaluated severe patients with pusher behavior and USN using the “summed equation” of the integrated subjective postural vertical (ISPV) metric. Since the “ratio” cannot detect errors caused by the addition of visual and somatosensory information and is dependent on one of the modalities, the “sum equation” can be used to evaluate severe cases of pusher behavior and USN to determine which modality impairment is caused by which modality impairment or whether the impairment is bilateral. This can be carried out by using the “sum equation” in the case of severe cases of pusher behavior with USN.

In a previous study, virtual reality systems were reported to adjust for this bias, showing that multisensory adjustment of visual-somatosensory relationships improves smoothness during walking [22]. Furthermore, the integration of multiple factors, sensory information, and the composition of the elements necessary for the task, such as the execution of movement, is considered an important procedure in the rehabilitation process [23].

Thus, this study aimed to evaluate the visual-somatosensory influences of integration by assessing SPV with eyes closed or opened.

## 2. Materials and Methods

### 2.1. Study Design

**Figure 1:**
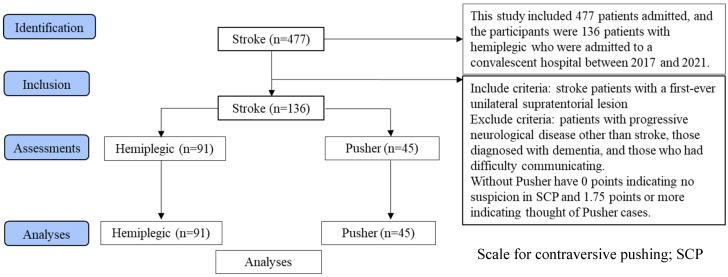
STROBE flow diagram of the study design.

This cross-sectional observational study was conducted in accordance with Strengthening the Reporting of Observational Studies in Epidemiology (STROBE) guidelines (Figure 1).

This study compared the pathophysiology of sensory integration in stroke hemiplegia cases and pusher cases with unilateral spatial neglect. The 136 stroke cases were assigned to the motor paralysis-only group and the pusher behavior group.

### 2.2. Participants

This study investigated 477 patients admitted to a convalescent hospital between 2017 and 2021, of whom 136 patients, including 91 patients with stroke hemiplegic and 45 patients with pusher behavior complicated by USN, were analyzed (Table 1). The inclusion criterion was stroke with the first unilateral supratentorial lesion. The exclusion criteria were progressive neurological diseases other than stroke, diagnosis of dementia, and the presence of communication difficulties. The study was conducted in accordance with the principles of the Declaration of Helsinki. An explanation of the study was provided to all patients, and informed consent was obtained via the opt-out method (no. Ryotokuji-00081). This study was approved by the University Hospital Medical Information Network Center (no. 000049806, approval on 31 March 2017), and all participants provided written informed consent.

### 2.3. Outcome Measurements

The demographic data examined included age, sex, days from onset, Brunnstrom recovery stage (BRS) of the lower limb [24], Scale for Contraversive Pushing (SCP) [25], and Behavioral Inattention Conventional Subtest (BITC) [26].

The BRS is a six-stage motor paralysis test that can assess voluntary function on the paretic side and the degree of damage to the corticospinal tract. The Functional Independence Measure (FIM) includes a battery of tests that assess usual ADLs [27]. The SCP can evaluate the severity of pusher behavior and is graded on a point scale ranging from 0 to 6, with 0 points indicating no suspicion of pusher behavior and 1.75 points or more indicating pusher behavior. The BITC evaluates behavioral neglect in the USN, with a score of less than 131 indicating unilateral spatial neglect.

The primary outcome of this study was the ratio or sum of SPV with eyes closed or opened: (1) SPV variability errors/SPV-EO variability errors as “ISPV”; (2) SPV variability errors + SPV-EO variability errors as “ISPVS”.

**Figure 2:**
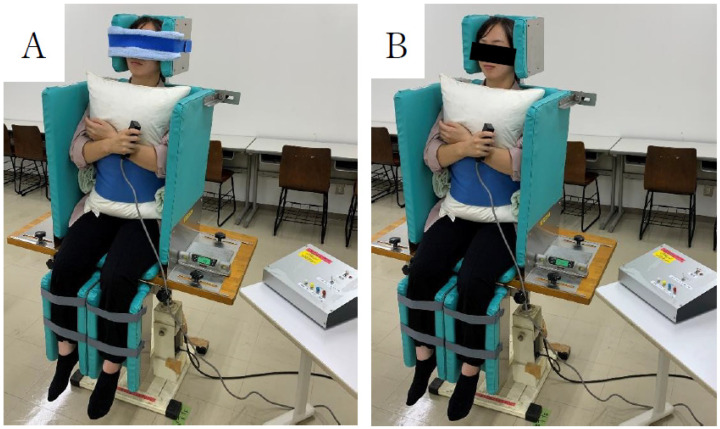
Assessment of postural vertical with eyes closed (**A**) and opened (**B**) on an automatic vertical board.

The SPV measurements were performed using a motorized tilt device. The mean of eight measurements was considered the directional error, while the standard deviation was considered the variability error (Figure 2) [20,21]. SPV was measured at the point where each participant was considered to sit vertically relative to the trunk line. The participants sat on the device and were inclined 15–20° to the left or right at a rate of 1.5° per second. The SPV measurements were performed in a randomized order. The starting direction of the tilt measurements was left or right based on the ABBABAAB or BAABABBA sequences. The standard deviation of the eight measurements was used as the PV tilt variability [20,21].

Participants were assessed in the sitting position on a motorized tilt device. Measurements were concluded with a hand-held switch, and the examiner recorded readings with a digital angle meter.

### 2.4. Statistical Analysis

The demographic data for each group included age, sex, days since onset, lower limb BRS score, total FIM score at admission, SCP, and SPV. The SPV measurements were performed using a motorized tilting device. Statistical analyses were performed using the parametric and/or non-parametric methods (Wilcoxon rank-sum test, Mann–Whitney *U* test, or chi-square test after the Shapiro–Wilk test; SPSS version 26; *p* < 0.05). The required statistical sample size was calculated using the Wilcoxson rank sum test with α = 0.05, power 1 − *β* = 0.8, and an effect size of 0.8, yielding a required sample size of 42 patients (G*power, ver 3.1) [28].

## 3. Results

**Figure 3:**
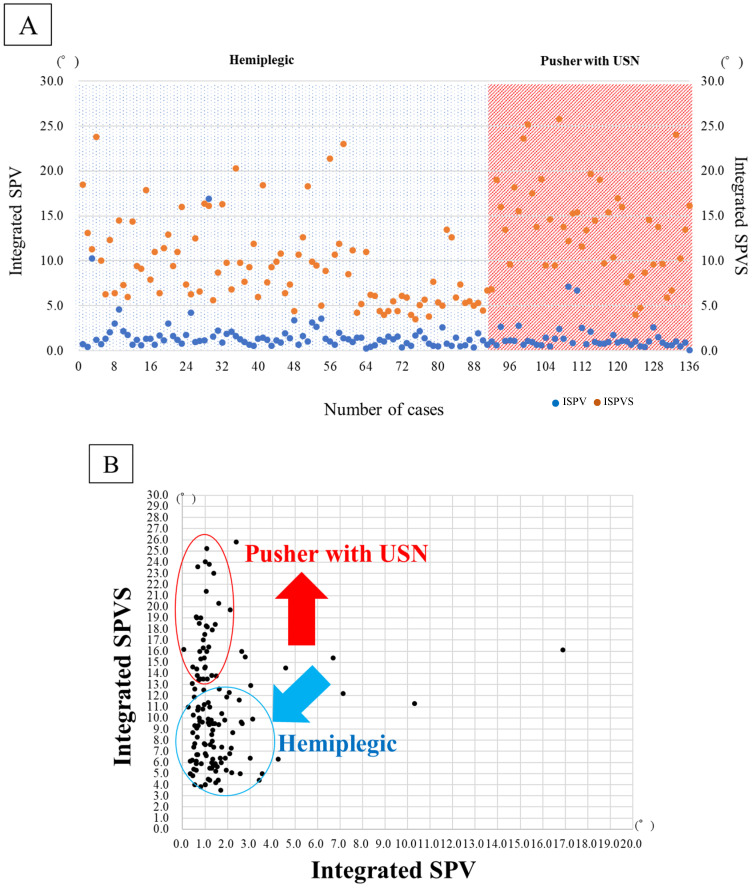

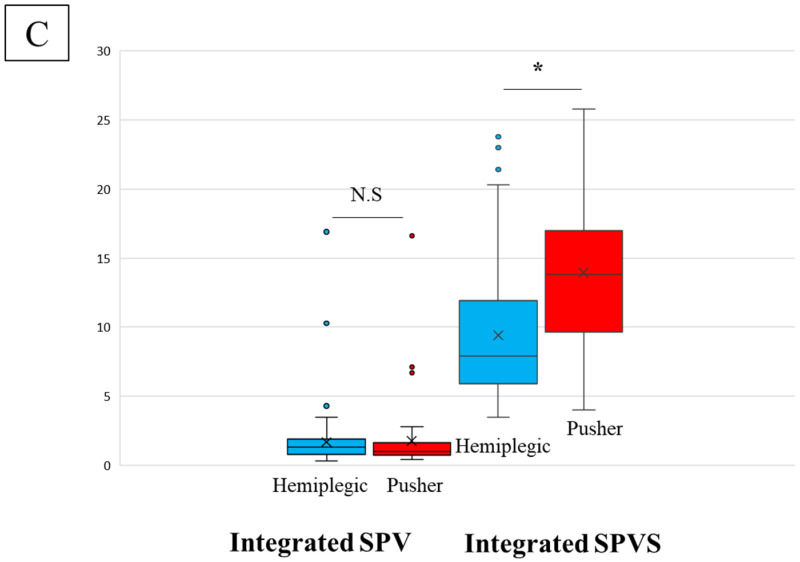
(**A**) Integrate SPV and Integrate SPVS in hemiplegic and patients with pusher behavior after stroke. (**B**) Deviations in Integrate SPV and Integrate SPVS in hemiplegic and patients with pusher behavior after stroke. (**C**) Comparisons of hemiplegic and pusher behavior with USN in Integrate SPV and Integrate SPVS. Integrated subjective postural vertical; Integrate SPV, Integrated subjective postural vertical sum; Integrate SPVS, unilateral spatial neglect; USN. N.S; no significant difference, *; significant difference (*p* < 0.05).

The demographic data of the 91 hemiplegic patients are presented in Table 1. They are mean age, 76.9 years; 47 males, 44 females; 35.6 days from onset; BRS of lower limb (I: 5, II: 44, III: 12, IV: 13, V: 11, VI: 6); total FIM score at admission of 62.5 points; ISPV of 1.64 (*p* = 0.70); ISPVS of 9.41° (*p* = 0.00, Figure 2). The corresponding data of the 45 patients with pusher behavior and USN were as follows: mean age, 72.9 years; 31 males, 14 females; 47.1 days from onset; BRS of lower limb (I: 4, II: 25, III: 9, IV: 3, V: 3, VI: 1), SCP of 4.6 ± 1.5 points; BITC of 73.6 points; total FIM score at admission of 40.6 points; ISPV of 1.35 (*p* = 0.70); and ISPVS of 13.96° (*p* = 0.00, Figure 3A,B).

In Figure 3A, both hemiplegic and pusher cases show SPV deviations, and there seems to be no difference in visual modality compensation in ISPV. However, pusher cases are also complicated by somatosensory deficits, and since there is no difference in quotient, the sum is calculated as in ISPVS. The difference clearly appears to be magnified.

In Figure 3B, the scatter plots suggest that pusher cases with unilateral spatial neglect have difficulty with integrated visual and somatosensory processing. Both visual integration and somatosensory modalities are impaired simultaneously.

In Figure 3C, statistically significant differences are observed in ISPVS. This suggests that the effect of higher brain dysfunction on sensory integration is more pronounced in patients with pusher behavior than in hemiplegic patients with motor paralysis alone.

The physical functions of patients with hemiplegic and pusher behaviors ranged from severe to mild, with no differences in ADLs. However, many pushers are known to experience severe brain dysfunctions. The results did not show any significant differences in demographic data or ISPV between the two groups of hemiplegic and pusher behavior and USN patients (Figure 3). However, the number of patients with USN was significantly higher than the number of patients with hemiplegic stroke in the ISPVS group (*p* < 0.05; Table 1, Figure 3C).

## 4. Discussion

Romberg’s characterization of visual modalities suggests that assessing reweighting and sensory integration, as well as pathophysiological interpretation and understanding, may be useful in setting rehabilitation goals. In this study, we investigated the influence of vision as a somatosensory modality on postural control, particularly in pushers.

The results showed no significant differences in demographic data or ISPV between the two groups. By contrast, the ISPVS of pusher behavior patients was significantly higher than that of hemiplegic patients (*p* < 0.05). This result indicates that ISPV was also lower when the SPV variability errors were low in eyes closed and open, reflecting the pathophysiological effects of vision as compensation for somatosensory perception. High values of SPV variability errors in closed and open eyes were also lower when the two modalities were divided by the value of the two modalities. The high values of the closed and open SPV variabilities were similarly low when the values of the two modalities were divided. Therefore, in these patients, SPV mobility was high, but there was an error in the integration process. However, a low ISPV may indicate a misinterpretation of the original pathophysiology. Therefore, a high ISPV value was reflected in the ISPVS, which is an “additive process”. In other words, it is possible to capture the pathological characteristics of a disorder through the integrated processing of modalities.

Overall, ISPV is a parameter that evaluates the ability of vision to correct somatosensory modalities by dividing the SPV variability into closed and open eyes. Vision is a cognitive process in which the eyes move at a rate of 50 ms, with perception occurring at 150 ms [29]. Visual input is considered to delay the cognitive process by ≥200 ms, and Farshchiansadegh et al. (2015) reported a significant reduction in processing errors in motor learning for spatial recognition under conditions of a 300 ms delay [30]. This mechanism involves environmental integration of the body’s internal and external environments through sensory processing in the visual cortex to the temporal parietal region. In addition, motor execution is monitored as a feedback mechanism to ensure consistent integration of visual and somatosensory processes. It has been well-established that assessing the “ratio” between sensory modalities in the SPV test is a key factor in the “cognitive process”. The evaluation of the “ratio” between sensory modalities on the SPV test, as in the conventional Romberg test, is believed to evaluate the degree of dependence on vision in postural control.

The ISPVS “sum” of the SPV variability in the eyes closed and opened positions indicates the “sum” of the error itself between the visual and somatosensory modalities. Therefore, we believe that it is possible to evaluate the ability to modify the ability to control posture using visual and somatosensory modalities. For the ISPVS, the “sum” principle allows us to consider this sum to be an indicator of visuospatial and somatosensory perception.

The ISPV can evaluate “the ability to integrate and process the two subjective postural vertical sensory modalities of eyes closed and opened”, making it possible to know whether the patient can adjust the information of preferential or selective postural control to multiple sensory processes according to the purpose [31]. Overall, we found no difference in the ratio of the integrated processing ability of somatosensory and visual processing in the ISPV between patients with pusher behavior and USN compared to patients with hemiplegic after stroke. This suggests that the ISPVS is necessary to evaluate the magnitude of the “summary” of the visual and somatosensory processing in patients with pusher behavior USN. The ISPV was significantly higher in USN pushers than in hemiplegic patients. Visual perception may not compensate for postural control modifications when postural control impairment occurs with impaired visuospatial perception, as evaluated against the increased influence of USN [32,33,34].

Sensory modality impairment impairs the ability to integrate or coordinate postural and motor control, which in turn impairs the ability to coordinate and integrate the senses for postural control or task performance. Furthermore, the resulting impairment in sensory integration has been shown to promote the fear of falling [35]. Patients are prioritized as an element of intervention because they are considered more likely to present with balance disorders during integrated processing. Balance impairments are hierarchical in function, and the integration process is an important component. Verticality is implemented early during rehabilitation among balance abilities. Visuospatial and kinesthesia are known to lead to learning as a result of the FIM comparison, which is an important finding of this study. It is perceived during the walking motion and plays a role in the smoothness of motion and purpose in the visual field. It also influences motor learning and can be a useful source of information for ADL situations. Adaptation occurs through the integration of sensory modalities. Patients with USN are characterized by the specific pathophysiology of a two-modality disorder with visual and somatosensory deficits. In contrast, sensory modalities have been found to optimally coordinate postural control, which requires multisensory integration [36]. This suggests that the impairment of diverse modalities may occur alone or during integration.

This study had some limitations. First, it was a cross-sectional study. In future studies, it will be necessary to follow the course and progression of the disease using rehabilitation interventions. Second, further research is needed to examine and clarify the impact of the ISPV on the functional prognosis of stroke survivors.

## 5. Conclusions

Overall, the results of this study suggest that there are no differences in the proportion of somatosensory and visually integrated processing abilities in the ISPV. However, the proportion of pushers in the ISPVS group was significantly higher than that in stroke patients with hemiplegic. This study provides important insights into the pathophysiological characteristics of patients with pusher syndrome and USN in the field of rehabilitation medicine.

## Figures and Tables

**Table 1:** Demographic data in a stroke of hemiplegic and in patients with pusher and unilateral spatial neglect.

Variables	Hemiplegic (n = 91)	Pusher (n = 45)	*p*-Value
Age ^a^	68.8 ± 13.5	72.9 ± 10.0	0.30
Sex ^c^	Men: 34	Men: 31	0.02
	Women: 57 *	Women: 14	
From on set ^a^	35.7 ± 37.3	47.1 ± 29.8 *	0.00
Stroke type ^c^	CI: 43	CI: 22	0.60
	CH: 48	CH: 23	
BRS Lower limb ^b^	Ⅰ: 5	Ⅰ: 4	0.00
	Ⅱ: 43 *	Ⅱ: 25	
	Ⅲ: 13	Ⅲ: 10	
	Ⅳ: 13	Ⅳ: 2	
	Ⅴ: 11	Ⅴ: 3	
	Ⅵ: 6	Ⅵ: 1	
SCP ^b^	0.1 ± 0.3	4.6 ± 1.5 *	0.00
USN	4	40	-
BITC	93.0 ± 24.0	73.6 ± 42.1	-
Lesion side ^c^	Right: 58 *	Right: 36	0.00
	Left: 33	Left: 9	
Legion site	ACA: 25	ACA: 7	-
	MCA: 13	MCA: 16	
	Corona radiata: 11	Corona radiata: 5	
	Thalamus: 21	Thalamus: 9	
	Putamen: 21	Putamen: 8	
Admission FIM ^a^	62.4 ± 24.5 *	40.6 ± 16.6	0.00
SPV directional errors ^a^	−0.2 ± 2.5	−0.1 ± 3.4	0.78
SPV-EO directional errors ^a^	0.1 ± 1.9	−0.6 ± 2.4	0.07
SPV variability errors ^a^	5.2 ± 3.0	7.6 ± 3.6 *	0.05
SPV-EO variability errors ^a^	4.2 ± 2.5	6.5 ± 3.2 *	0.01
ISPV ^a^	1.7 ± 2.0	1.8 ± 2.6	0.70
ISPVS ^a^	9.4 ± 4.7	14.0 ± 5.2 *	0.00

^c^ CI, cerebral infarction; ^c^ CH, cerebral hemorrhage; ^b^ BRS, Brunstrom recovery stage; ^b^ SCP, Scale for Contraversive Pushing; USN, unilateral spatial neglect; BITC, Behavioral Independence Test conventional test; ^a^ SPV-EO, subjective postural vertical with eyes opened; ^a^ ISPV, integrated subjective postural vertical; ^a^ ISPVS, integrated subjective postural vertical sum; ^a^ FIM, functional independence measure. ^a^ Wilcoxon rank-sum test, ^b^ Mann-Whitney U test, ^c^ Chi-square test: * significant difference (*p* < 0.05).

## Data Availability

The original contributions presented in the study are included in the article, further inquiries can be directed to the corresponding author.

## References

[1] Ministry of Health, Labour and Welfare (2020). “Vital Statistics”, Vital, Health and Social Statistics Office to the Director-General for Statistics and Information Policy.

[2] Ministry of Health, Labour and Welfare (2020). Overview of the System and the Basic Statistics.

[3] Brott T., Adams H.P., Olinger C.P., Marler J.R., Barsan W.G., Biller J., Spilker J., Holleran R., Eberle R., Hertzberg V. (1989). Measurements of acute cerebral infarction: A clinical examination scale. Stroke.

[4] Quinn T.J., Dawson J., Walters M.R., Lees K.R. (2009). Reliability of the modified Rankin Scale: A systematic review. Stroke.

[5] Folstein M.F., Folstein S.E., McHugh P.R. (1975). “Mini-Mental State”: A practical method for grading the cognitive state of patients for the clinician. J. Psychiatr. Res..

[6] Karnth H.-O. (2001). New insights into the functions of the superior temporal cortex. Nat. Rev..

[7] Manso A., Ganaca M.M., Caovilla H.H. (2016). Vestibular rehabilitation with visual stimuli in peripheral vestibular disorders. Braz. J. Otorhinolaryngol..

[8] Corriveau H., Hébert R., Raîche M., Prince F. (2004). Evaluation of Postural Stability in the Elderly with Stroke. Arch. Phys. Med. Rehabil..

[9] Shumway-Cook A., Woollacott M. (2000). Attention Demands and Postural Control: The Effect of Sensory Context. J. Cerontolngt. Med. Sci..

[10] Ionta S., Heydrich L., Lenggenhager B., Mouthon M., Fornari E., Chapuis D., Gassert R., Blanke O. (2011). Multisensory Mechanisms in Temporo-Parietal Cortex Support Self-Location and First-Person Perspective. Neuron.

[11] Raiser T.M., Flanagina V.L., Duering M., Ombergena A.V., Ruehl R.M., Eulenburg P.Z. (2020). The human corticocortical vestibular network. NeuroImage.

[12] Liu B., Zhao G., Jin L., Shi J. (2021). Association of Static Posturography With Severity of White Matter Hyperintensities. Front. Neurol..

[13] Horak F.B. (2006). Postural orientation and equilibrium: What do we need to know about neural control of balance to prevent falls. Age Ageing.

[14] Berge J.E., Goplen F.K., Aarstad H.J., Storhaug T.A., Nordahl S.H.G. (2022). The Romberg sign, unilateral vestibulopathy, cerebrovascular risk factors, and long-term mortality in dizzy patients. Front. Neurol..

[15] Romberg M.H. (1846). Lehrbuch der Nervenkrankheiten des Menschen.

[16] Romberg M.H. (1851). Lehrbuch der Nervenkrankheiten des Menschen.

[17] Zhong X., Yost W.A. (2013). Relationship between Postural Stability and Spatial Hearing. J. Am. Acad. Audiol..

[18] Lê T.T., Kapoula Z. (2008). Role of ocular convergence in the Romberg quotient. Gait Posture.

[19] TjernstrÖm F., BjÖrklund M., MalmstrÖm E.M. (2015). Romberg ratio in quiet stance posturography-Test to retest reliability. Gait Posture.

[20] Sawa K., Amimoto K., Ishigami K., Miyamoto T., Ishii C., Suzuki R., Tamura M., Morizane A., Komatsu C., Miyagami M. (2023). Recovery process of vertical perception and activities of daily living in stroke patients: A retrospective cohort study. Brain Behav..

[21] Sawa K., Amimoto K., Ishigami K., Miyamoto T., Setoyama C., Suzuki R., Nozomi K., Tamura M., Miyagami M. (2022). Efficacy of lateral truncal tilt training with a wedge on postural vertical and activities of daily living in recovery phase after stroke: A randomized crossover trial. Neuro Rehabil..

[22] Rothacher Y., Nguyan A., Lenggenhager B., Kunz A., Brugger P. (2018). Visual capture of gait during redirected walking. Sci. Rep..

[23] Yelnik A.P., Tasseel Ponche S., Andriantsifanetra C., Provost A.C., Rougier C. (2015). Walking with eyes closed is easier than walking with eyes open without visual cues: The Romberg task versus the goggle task. Ann. Phys. Rehabil..

[24] Brunnstrom S. (1970). Movement Therapy in Hemiplegia. A Neurophysiological Approach.

[25] Karnath H.O., Ferber S., Dichgans J. (2000). The origin of contraversive pushing: Evidence for a second graviceptive system in humans. Neurology.

[26] Ishiai S. (1999). Behavioural Inattention Test.

[27] Granger C.V., Hamilton B.B., Keith R.A., Zielezny M., Sherwin F.S. (1986). Advances in functional assessment for medical rehabilitation. Top. Geriatr. Rehabil..

[28] Faul F., Erdfelder E., Buchner A., Lang A.G. (2009). Statistical power analyses using G*Power 3.1: Tests for correlation and regression analyses. Behav. Res. Methods.

[29] Scott S.H., Cluff T., Lowrey C.R., Takei T. (2015). Feedback control during voluntary motor actions. Curr. Opin. Neurobiol..

[30] Farshchiansadegh A., Ranganathan R., Casadio M., Mussa-Ivaldi F.A. (2015). Adaptation to visual feedback delay in a redundant motor task. J. Neurophysiol..

[31] Karmali F., Bermúdez Rey M.C., Clark T.K., Wang W., Merfeld D.M. (2017). Multivariate Analyses of Balance Test Performance, Vestibular Thresholds, and Age. Front. Neurol..

[32] Rode G., Pagliari C., Huchon H., Rossetti Y., Pisella L. (2017). Semiology of neglect: An update. Ann. Phys. Rehabil. Med..

[33] Pimenta C., Correia A., Alves M., Virella D. (2017). Effects of oculomotor and gaze stability exercises on balance after stroke: Clinical trial protocol. Porto. Biomed. J..

[34] Jahn K., Strupp M., Krafczyk S., Schüler O., Glasauer S., Brandt T. (2002). Suppression of eye movements improves balance. Brain.

[35] Schinkel-Ivy A., Inness E.L., Mansfield A. (2016). Relationships between fear of falling, balance confidence, and control of balance, gait, and reactive stepping in individuals with sub-acute stroke. Gait Posture.

[36] Horak F.B., Nashner L.M., Diener H.C. (1990). Postural strategies associated with somatosensory and vestibular loss. Exp. Brain Res..

